# Peptide-modified Substrate for Modulating Gland Tissue Growth and Morphology *In Vitro*

**DOI:** 10.1038/srep11468

**Published:** 2015-06-22

**Authors:** Hiroaki Taketa, Gulsan Ara Sathi, Mahmoud Farahat, Kazi Anisur Rahman, Takayoshi Sakai, Yoshiaki Hirano, Takuo Kuboki, Yasuhiro Torii, Takuya Matsumoto

**Affiliations:** 1 Department of Biomaterials, Okayama University, 2-5-1 Shikata-Cho, Okayama, 700-8558, Japan; 2 Department of Oral-Facial Disorders, Osaka University, 1-8 Yamada-oka, Suita, 565-0871, Japan; 3 Department of Chemical Engineering, Kansai University, 3-3-1 Yamate, Suita 564-8680, Japan

## Abstract

*In vitro* fabricated biological tissue would be a valuable tool to screen newly synthesized drugs or understand the tissue development process. Several studies have attempted to fabricate biological tissue *in vitro*. However, controlling the growth and morphology of the fabricated tissue remains a challenge. Therefore, new techniques are required to modulate tissue growth. RGD (arginine-glycine-aspartic acid), which is an integrin-binding domain of fibronectin, has been found to enhance cell adhesion and survival; it has been used to modify substrates for *in vitro* cell culture studies or used as tissue engineering scaffolds. In addition, this study shows novel functions of the RGD peptide, which enhances tissue growth and modulates tissue morphology *in vitro*. When an isolated submandibular gland (SMG) was cultured on an RGD-modified alginate hydrogel sheet, SMG growth including bud expansion and cleft formation was dramatically enhanced. Furthermore, we prepared small RGD-modified alginate beads and placed them on the growing SMG tissue. These RGD-modified beads successfully induced cleft formation at the bead position, guiding the desired SMG morphology. Thus, this RGD-modified material might be a promising tool to modulate tissue growth and morphology *in vitro* for biological tissue fabrication.

Recent advancement of material sciences in combination with cell and molecular biology has increased the possibilities for *in vitro* biological tissue fabrication. Optic cup and adenohypophysis tissue, for example, have been fabricated by using embryonic stem cells encapsulated in a type I collagen gel^1^,^2^. Preliminary developmental stages of teeth and hair follicle formation have been engineered by using organotypic culture systems^3^,^4^,^5^. We have fabricated cartilage tissue mimicking endochondral ossification by using 3D constructs of mesenchymal stem cells *in vitro*^6^. Although artificially fabricated tissue contains fully differentiated cells distributed throughout the tissue and has protein expression profiles similar to that of native tissues, currently most artificial tissues are limited in size and displays incomplete morphology. Thus, *in vitro* manipulation of tissue growth and morphology still remains a challenge.

The development of a mouse submandibular gland (SMG) begins with the protrusion of epithelial tissue into mesenchymal tissue on embryonic day 11. The SMG morphological change including the formation of a number of buds and ductal elongation is called branching morphogenesis^7^. This specific morphological change can also be seen in the development of other organs, such as lungs, kidneys, mammary glands, and spleen. A number of studies have investigated the mechanism of this morphological change and have demonstrated the importance of external stimuli. For example, growth factors such as various FGFs and EGF influence the up/down regulation of mitogen-activated protein kinase (MAPK), phospholipase Cγ1 (PLCγ1), and phosphatidyl-inositol-3-kinase (PI3K) in the regulation of SMG growth and morphology^8^,^9^,^10^. In addition, secreted matrix proteins play pivotal roles in SMG morphogenesis. Recent studies indicate that fibronectin expression is crucial for cleft formation in branching SMG tissue, which is associated with the conversion of cell-cell adhesions to cell-matrix adhesions in those regions^11^,^12^. Since the tripeptide sequence arginine-glycine-aspartic acid (RGD) was found in 1984 as the integrin-binding domain of fibronectin, this functional motif has been synthesized artificially and utilized as a bioactive molecule in bioengineering studies^13^. For example, a peptide-immobilized alginate scaffold with osteoblast and chondrocyte composites was transplanted onto the back of a mouse, resulting in ectopic formation of the growth plate^14^. In this context, RGD-modified materials acted as a substrate that supported cell adhesion and survival under *in vivo* conditions^15^. To our knowledge, however, the use of the RGD peptide for modulating *in vitro* tissue growth and morphology has not been investigated yet. We hypothesized here that an RGD-modified material would be effective for artificial modulation of SMG growth and morphogenesis. To test this hypothesis, in this study, *in vitro* SMG explant culture was carried out by using RGD-modified alginate hydrogels with two different shapes, sheets and beads.

## Results

### SMG growth on RGD-modified gel sheets

**Figure 1:**
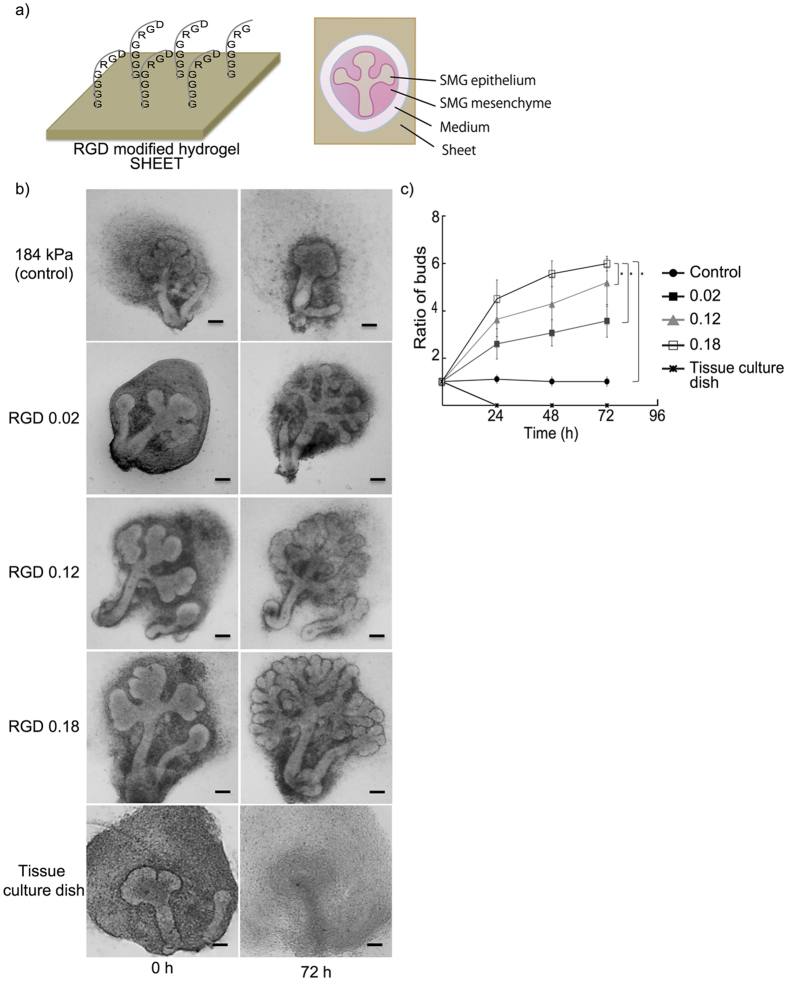
**a**) Schematic illustration of submandibular gland tissue (SMG) culture on hydrogel. **b**,**c**) SMG cultured on hydrogel sheets modified by introducing various RGD concentrations (0–0.18 mol/l), and SMG cultured on a tissue culture dish without any hydrogel substrate as a negative control (Bar = 50 μm). A stiffer gel sheet normally attenuates SMG growth, but the SMG growth was enhanced when RGD was introduced. The SMG growth changed in accordance with the introduced amount of RGD (*p < 0.05). Whereas, SMG was completely dissociated when cultured on the tissue culture dish.

Previously, we carried out an SMG organotypic culture on alginate hydrogel sheets with varying mechanical stiffness and obtained results indicating that bud expansion and cleft formation in the SMG were enhanced on the softer gel (4 kPa), but was attenuated on the stiffer gel (184 kPa)^16^. The stiffer gel was therefore used as a negative control for this study to evaluate the effect of RGD on the growth and morphogenesis of SMG tissue. We fabricated sheets of 184 kPa alginate hydrogels with different amounts of RGD and placed SMG tissue extracted from E12.5 ICR mouse embryos on them (Figure S1). The results indicated that RGD-modified alginate hydrogels enhanced the bud expansion and cleft formation of SMGs even when they were cultured on the stiffer gel. The number of buds in SMG tissue increased with rise in RGD (Fig. 1). A similar phenomenon was observed when SMG was cultured on RGD modified 4 kPa (soft) hydrogel (Figure S2). We also cultured SMG tissue on a conventional tissue culture dish to confirm that the enhanced tissue growth was linked to the presence of RGD. Strikingly, SMG tissue branching was not observed. The tissue appeared dissociated, although high cell adhesion to the tissue culture dish was seen under this condition (Fig. 1). Since the strongest effect of RGD was observed with the use of 0.18 wt% modified hydrogel, introduction of RGD at a 0.18 wt% was used in further experiments for this study.

### Possible mechanism for the observations

To understand the effect of RGD on epithelial tissue growth, epithelial tissue isolated from whole SMG tissue was cultured on RGD-modified alginate hydrogel sheets. The epithelial tissue cultured solely on RGD-modified alginate hydrogels did not grow but degenerated (Figure S3).

**Figure 2:**
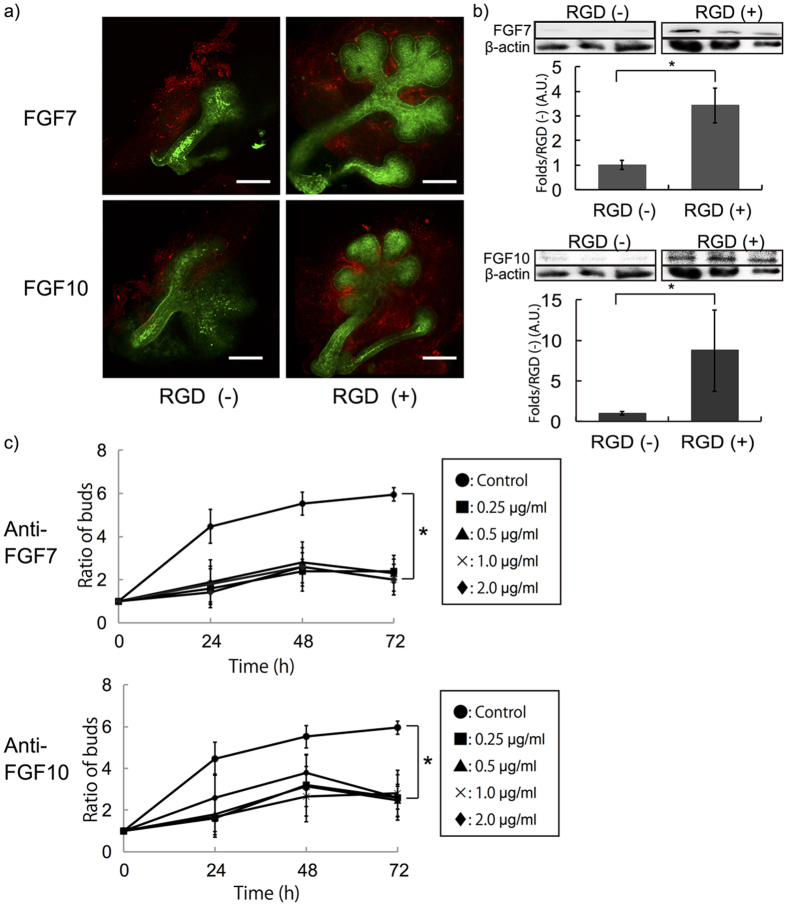
**a**) Immunofluorescent staining of FGF7 and FGF10 expressed in SMG tissue cultured for 24 hours on hydrogel sheets with and without RGD modification (Red: anti-FGF7/anti-FGF10, Green: PNA, Bar = 100 μm). **b**) Western **b**lotting results of FGF7/FGF10 expression in SMG tissue cultured for 72 hours. Higher expression of both proteins was detected in SMG cultured on RGD modified gel sheet. ß-actin used as control. **c**) Addition of anti-FGF7 or anti-FGF10 clearly attenuates SMG growth cultured on RGD modified gel sheet in accordance with the amount of antibodies. (*p < 0.05).

Immunofluorescent staining of FGF7 and FGF10, two important molecules involved in epithelial growth of SMG tissue, was carried out after 24 hours. The regions of expression of these proteins in SMG tissue did not change in relation to the presence of RGD, and both of these growth factors were expressed mainly in mesenchymal tissue (Fig. 2a). Quantitative results obtained by Western blotting analyses indicated significantly high expression of these growth factors in SMG cultured for 72 hours on RGD-modified gels (Fig. 2b). To further confirm the roles of these growth factors, antibodies to FGF7 and FGF10 were applied to the SMG culture on the RGD-modified gel sheet. Strikingly, SMG growth was attenuated even when cultured on an RGD-modified hydrogel sheet (Fig. 2c).

**Figure 3:**
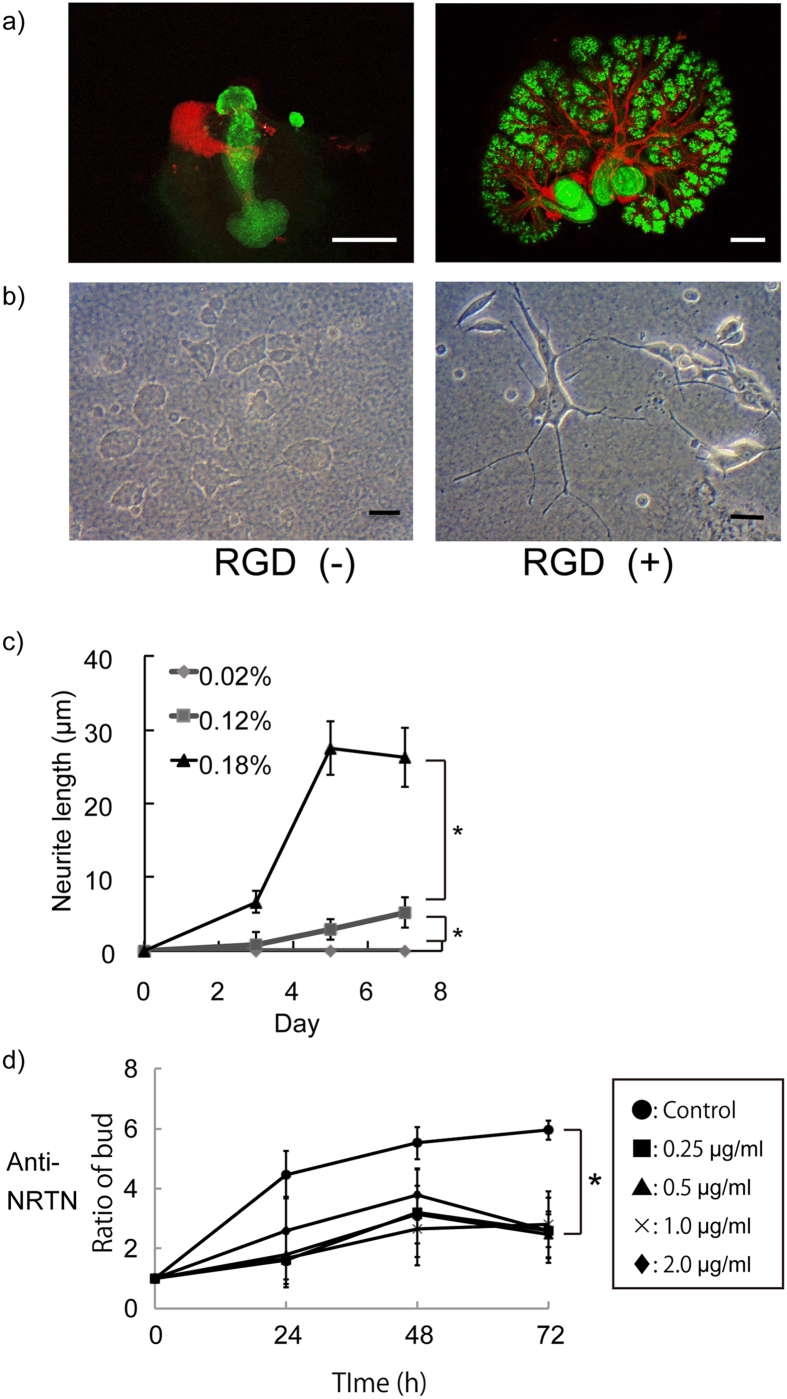
**a**) Neural growth in SMG tissue cultured for 72 hours on hydrogel sheets with and without RGD modification. Neural expansion is enhanced on the RGD-modified sheet (Red: anti-βIII tubulin, Green: PNA, Bar = 100 μm). **b**) Neural cell culture for 7 days on hydrogel sheets with and without RGD modification. Cells on the RGD-modified sheet show better neurite growth than do cells on the sheet without RGD modification (Bar = 20 μm). **d**) Addition of anti-neurturin clearly attenuates SMG growth according to the amount of antibody. (*p < 0.05).

Immunofluorescent staining of βIII tubulin was used to detect neuronal innervation and peanut agglutinin (PNA) for epithelium in SMG tissue cultured on gel sheets with or without RGD modification for 72 hours. The results indicated a magnificent distribution of neural tissue in the SMG cultured on the RGD-modified gel sheet whereas non-growing, localized neuronal tissue was observed in SMG cultured on the unmodified gel sheet (Fig. 3a). To confirm the effect of RGD on neurite outgrowth, PC12 cells were cultured for 7 days on alginate hydrogel sheets with or without RGD modification. The neuronal growth of PC12 cells was dramatically enhanced when the cells were cultured on a substrate with RGD (Fig. 3b,c). Moreover, antibody to neurturin (NRTN), a neurotrophic factor, was used on the SMG cultures growing on RGD-modified gel sheets. Strikingly, the SMG growth was attenuated even when it was cultured on an RGD-modified hydrogel sheet in the presence of this antibody (Fig. 3d).

### Effect of local application of RGD-modified beads on SMG growth

**Figure 4:**
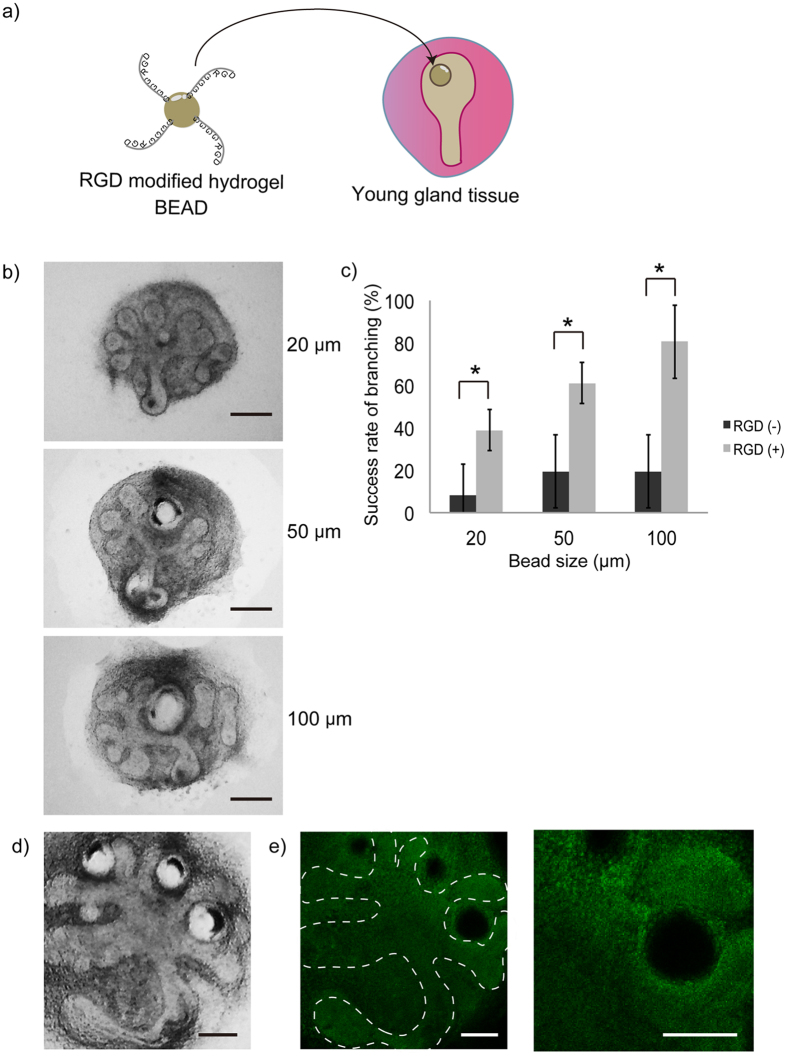
**a**) SMG tissue cultured with RGD modified hydrogel beads of different sizes. The ratio of cleft formation changed in accordance with the bead size (Bar = 100 μm). **b**) For beads of any size, the rate of successful cleft formation was higher for RGD-modified beads. **c**) The beads placed on SMG tissue triggered cleft formation locally (Bar = 50 μm). **d**) Ki67 staining indicated the proliferation of both epithelial and mesenchymal cells around the beads (Bar = 50 μm). (*p < 0.05).

Next, to investigate the effect of localized application of RGD-modified substrate, hydrogel beads (20–100 μm) with or without RGD modification were prepared^17^ (Figure S4). Unmodified control gel beads of different sizes were placed on isolated SMG and cultured for 72 hours on a control stiffer gel sheet. The results indicated that cleft formation sometimes occurred at the bead position. The ratio of cleft formation at the bead site increased with increasing bead size, but it was still ≤19%. Next, RGD-modified gel beads of various sizes were applied. The application of these beads dramatically enhanced the ratio of cleft formation by about 38.9% for 20 μm beads, 61.1% for 50 μm beads, and 80.6% for 100 μm beads, which was four times higher than the that with the control gel beads (Fig. 4a–c, and Figure S5). For further confirmation of the possible use of RGD-modified hydrogel beads for modulating tissue morphogenesis, three RGD-modified beads were placed on the cultured SMG. The results indicated the possibility of controlling cleft formation and morphology of the SMG tissue as previously predicted (Fig. 4d). Ki67 staining of SMG tissue, cultured in this condition, was carried out to evaluate cellular proliferation. The results indicated that both epithelial and mesenchymal cells proliferated specifically at the region surrounding the bead on the SMG tissue (Fig. 4e).

## Discussion

Our previous work indicated that RGD modified substrates have the potential to enhance SMG tissue growth^16^. To confirm that this phenomenon is indeed dependent on the presence of RGD, we prepared RGD-modified alginate hydrogel sheets with different concentrations of RGD and cultured SMG tissue on it. Strikingly, the bud expansions and cleft formations of cultured SMG increased according to the amount of RGD present. Also, non-modified RGD substrate did not lead to SMG growth, suggesting that RGD is the key factor in this phenomenon.

SMG tissue consists of epithelial and mesenchymal tissues, and the mesenchymal tissue in the SMG surrounds the epithelial tissue. Previous studies have indicated that exogenous FGFs play crucial roles in SMG morphogenesis^16^. FGF7 induces epithelial budding, FGF10 induces ductal elongation, and both are inhibited by fibroblast growth factor receptor (FGFR) or extracellular signal-regulated kinase 1/2 (ERK1/2) signalling inhibitors^18^,^19^,^20^. In this study, it was found that both FGF7 and FGF10 were highly expressed in SMG tissue cultured on RGD-modified gels. The antibodies to these growth factors attenuated SMG growth when it was cultured on RGD-modified gels. These results are supported by previous studies reporting similar effects with these antibodies^16^. These results suggest that RGD affects the up-regulation of FGF7 and FGF10 expression in SMG cultures.

A previous report revealed that neural development, especially the neurite outgrowth from the parasympathetic ganglion (PSG) in the SMG tissue, is critical in SMG development^21^. In the present study, the neuronal network in the SMG cultured on RGD-modified gels was well distributed. Also, a PC12 neuronal cell line showed a remarkable neurite growth on this RGD-modified gel. This finding is consistent with previous studies indicating a robust neurite outgrowth of PC12 cells on fibronectin-coated substrates^22^. Antibody to NRTN, a neurotrophic factor, inhibited SMG growth even on the RGD-modified gel. Also, RGD-modified gels did not enhance bud expansions of epithelial cells isolated from the SMG tissue. All together, these results suggest that the RGD-modified alginate hydrogel sheet promoted growth of SMG by enhancing neuronal growth in the SMG mesenchyme, as well as promoting increased expression of growth factors.

In the work described in the first part of this paper, SMG tissue culture was carried out on gels having a sheet morphology. RGD was present on the entire surface of the alginate hydrogel sheets and affected the entire SMG tissue under these conditions. Therefore, to investigate the local effect of RGD, RGD-modified alginate hydrogel beads were used. The application of the RGD-modified beads dramatically enhanced the ratio of cleft formation four times than that with the non-RGD-modified gel beads. Moreover, SMG morphology was manipulated by placing the beads in a specific position on the SMG. We surmised that this phenomenon is caused by an increased local cell number in the region surrounding the bead because RGD promotes cell proliferation^23^. This hypothesis was confirmed by Ki67 staining, indicating that specific Ki67 positive cells were present around the bead. This bead-guided cleft formation in the SMG tissue resembles normal SMG development^24^.

A biomimetic environment is an artificial environment that reproduces the biological stimuli occurring within one’s body. Owing to the advancement of biomaterials science and tissue engineering, researchers are able to use biocompatible materials to make biomimetic environments *in vitro*^25^,^26^. RGD-modified materials have been utilized to improve cellular compatibility and enhance cell survival for cell therapy and tissue engineering^27^,^28^,^29^. In this study, RGD-modified alginate hydrogels with two different shapes were used for *in vitro* SMG tissue culture. RGD-modified hydrogels with sheet morphology promoted whole SMG tissue growth by increasing bud expansion as well as cleft formation. Furthermore, RGD hydrogels with a small-bead morphology acted locally, initiated cleft formation in the tissue, and allowed the growth of SMGs with the desired morphology. Thus, in this study, we clearly showed the potential of RGD for *in vitro* modulation of tissue growth and morphology.

## Conclusions

RGD can modulate *in vitro* tissue growth and morphology in both wide and localized areas. This RGD-based material should be a promising tool to manipulate *in vitro* tissue growth and morphology. Also, this *in vitro* SMG tissue growth modulation system can have a variety of uses including tissue arrays for drug screening and as a biologic tool to understand tissue development.

## Methods

### Preparation of alginate hydrogel sheets and beads

We previously found that a stiffer gel (with a Young’s modulus of 184 kPa) attenuates *in vitro* SMG growth^16^. Hence, in this study, we used this stiffer hydrogel as a control to evaluate the effect of the synthesized material on SMG tissue growth. A sodium alginate solution (0.3–4 wt%, Wako Pure Chemical, Japan) was poured into a mold made of a porous alumina plate. The mold was soaked in calcium chloride solution (5 wt%) for 1.5 hours to obtain the alginate gel sheet. The resultant alginate hydrogel sheet was washed with then stored in Dulbecco’s modified Eagle’s medium /Nutrient Mixture F-12 (DMEM-F12) medium (Wako Pure Chemical, Japan) supplemented with 1% penicillin/streptomycin (Nacalai Tesque, Japan) (DMEM-F12/PS) for at least 24 h and subsequently cut into small pieces for cell and organ culture (10 × 10 x 1.5 mm). Carbodiimide chemistry was utilized to fabricate a Gly-Gly-Gly-Gly-Arg-Gly-Asp (G4RGDS) conjugated alginate solution used to prepare the RGD modified alginate gels. One-ethyl-(dimethylaminopropyl)-carbodiimide (EDC, 1 mmol/l) and the co-reactant N-hydroxysulfosuccinimide (sulfo-NHS, 1 mmol/l) were made to react with 0.15 wt% of the alginate solution in a MES buffer (0.1 mol/l MES, 0.3 mol/l NaCl, pH 6.5) for 5 min to form a stable intermediate. The desired concentrations of peptide were then added to the solution and left to react overnight at room temperature. The peptide-modified alginate was purified by dialysis and lyophilized for storage^14^.

Microbeads were prepared as previously described using a 3D-printing device^17^. Four wt% RGD modified or non-modified alginate solutions were used. A syringe pump was used to supply the alginate solution at a flow rate of 500 μl/min. Alginate gelation was performed with a CaCl_2_-oleic acid solution. The bead size was controlled by stirrer speed. To obtain 20 to100 μm sized beads, the stirrer speed used was 1250 to 500 rpm, respectively (Figure S4). After the beads were prepared, they were left for 5 min to allow precipitation to occur. Then, the CaCl_2_ solution was aspirated off, and the beads were collected. Excess CaCl_2_ was removed by centrifuging the beads at 1000 rpm for 2 min. The beads were then washed 3 times with phosphate buffered saline (PBS), centrifuging at 1000 rpm for 3 min in between washes.

### SMG tissue and cell culture

All animal procedures undertaken in this study were strictly in accordance with the “Guidelines for Animal Experiments at Okayama University” and all experimental protocols were approved by Okayama University (OKU-2013033). SMG tissue extracted from E12.5 ICR mouse embryos was placed directly on non-modified alginate hydrogel sheets or the RGD-immobilized alginate hydrogel sheets with a mechanical stiffness of 4 kPa or 184 kPa, or alternatively on tissue culture dishes. The SMG culture was carried out at 37 °C in a humidified 5% CO_2_ atmosphere. DMEM-F12/PS medium was used for this organ culture. The concentration of chemicals used in this study was determined from previous reports^17^,^30^. Antibodies that were used are anti-FGF7 (0.25–2.0 μg/ml, AB Biotech, CA), anti-FGF10 (0.25–2.0 μg/ml, Millipore, CA), and anti-neurturin (0.25–2.0 μg/ml, R&D systems, MN). The soft hydrogel promotes SMG tissue growth, while the stiff hydrogel attenuates SMG tissue growth in *in vitro* culture system. Therefore, when we needed to evaluate the inhibitory effect of the chemicals, we used the soft hydrogel as a positive control. On the other hand, when we needed to evaluate the accelerator effect of the RGD peptide, stiffer alginate hydrogels without RGD modification were used as a negative control. A microscope (TE-2000, Nikon, Japan) was used to observe the growth and morphological changes of the cultured SMG. The bud number of the growing SMG in the images was measured at the time points 0, 24, 48, and 72 hours. For further modulation of SMG morphogenesis, alginate hydrogel beads (20–100 μm) with or without RGD modification were placed in close proximity to the SMG epithelial tissue cultured on stiffer hydrogel sheets. PC12 cells (ATCC, VA) were grown in DMEM supplemented with 10% horse serum, 5% fetal bovine serum, and 1% penicillin/streptomycin (DMEM/HS/FBS/PS) (Life Technologies, NY) in a 5% CO_2_ humidified atmosphere at 37 °C. A microscope (TE-2000, Nikon) was used to observe the growth and morphological changes of the cultured SMG. The SMG bud number was counted at 0, 24, 48 and 72 hours. The counting was performed by three individuals in a controlled, blinded fashion. The initial number of buds at 0 hours was considered as 1. The bud number at each time point was divided by the bud number at 0 hours to get a ratio. For cleft initiation using beads, new cleft formation was defined as occurring when divided epithelium growth exceeded the bead position in both the X and Y axis. For the neurite growth study, five thousand cells were seeded on the hydrogel and cultured in DMEM/HS/FBS/PS medium containing 50 ng/ml of nerve growth factor (NGF, Millipore). Total neurite length in individual cells was measured using Image J software (NIH, MD)^31^.

### Immunofluorescence staining and Western blotting

The cultured SMGs were fixed with 4% paraformaldehyde (PFA) and incubated with fluorescein isothiocyanate (FITC)-conjugated PNA (1:200, Sigma-Aldrich, MO), anti-βIII-tubulin (1:1000, R&D Systems), anti-FGF7 (1:1000, AB Biotech), anti-FGF10 (1:2000, Millipore), or anti-ki-67 (1:1500, Abcam, UK). The antibodies were detected using Alexa Fluor 488 or 568 conjugated secondary antibodies (Life Technologies). Images were obtained using confocal microscopy (C1, Nikon). The total amount of protein obtained from the cultured tissue was calculated using a BCA protein assay kit (Pierce Biotechnology, IL) and then the following antibodies were used in Western blot analysis: anti-FGF7 (1:1000) and anti-FGF10 (1:2000). Goat anti-rabbit horseradish peroxidase conjugated secondary antibody (1:8000, Santa Cruz Biotec, CA) was used as a secondary antibody. ß-Actin antibody (1:5000, Abcam) was used as a loading control. Western blot data were quantified by comparing the relative density of target bands to the loading control by using Image J software. Additional information is supplied in the Supplementary data.

### Statistical analysis

Quantitative tests were conducted in quadruplicate, and mean values with standard deviations were then calculated. Statistical significance at the 5% level was evaluated using one-way analysis of variance (ANOVA) with Scheffe’s F test.

## Additional Information

**How to cite this article**: Taketa, H. *et al*. Peptide-modified Substrate for Modulating Gland Tissue Growth and Morphology *In Vitro*. *Sci. Rep*. **5**, 11468; doi: 10.1038/srep11468 (2015).

## Supplementary Material

Supplementary Information
