# Supplementary material for: Peptide-modified Substrate for Modulating Gland Tissue Growth and Morphology In Vitro
Source: Sci Rep. 2015 Jun 22;5:11468. doi: 10.1038/srep11468 (PMC4476418; doi:10.1038/srep11468)
Supplement: Supplementary Information [file srep11468-s1.doc]

Supplementary data

**Peptide-modified Substrate for Modulating Gland Tissue Growth and Morphology *In Vitro***

Hiroaki Taketa^a^, Gulsan Ara Sathi^a^, Mahmoud Farahat^a^, Kazi Anisur Rahman^a^, Takayoshi Sakai^b^, Yoshiaki Hirano^c^, Takuo Kuboki^a^, Yasuhiro Torii^a^, Takuya Matsumoto^a^,*

Supplementary method

***Western blot analysis:***

Total cell lysates of SMG tissue were prepared by adding x1 sampling buffer and boiled for 2 min in sodium dodecyl sulfate (SDS) gel-loading buffer (0.1M Tris-HCl, pH 6.8, 20% glycerol, 2.5% SDS, 0.5% bromophenol blue, and 5% β-mercaptoethanol). The total amount of protein obtained from the cultured tissue was calculated using a BCA protein assay kit (Pierce Biotechnology, IL). The protein sample were then loaded and separated by 10% SDS-PAGE and transferred onto polyvinylidene difluoride (PVDF) membranes (Millipore, MN) using Mini Trans-Blot® system (Bio-Rad, Hercules, CA). The membranes were incubated with primary antibodies overnight at 4ºC, followed by addition of secondary antibodies for 1hour at room temperature. As a control ß-actin was used. Proteins were then visualized using Luminata Forte Western HRP substrate (Millipore) and a CCD-type imager (Image Quant LAS 4000 mini, GE Healthcare, UK) according to the manufacturer's instructions. The antibodies used for both immunofluorescences and western blot are listed in supplementary table 1.

Supplementary figures


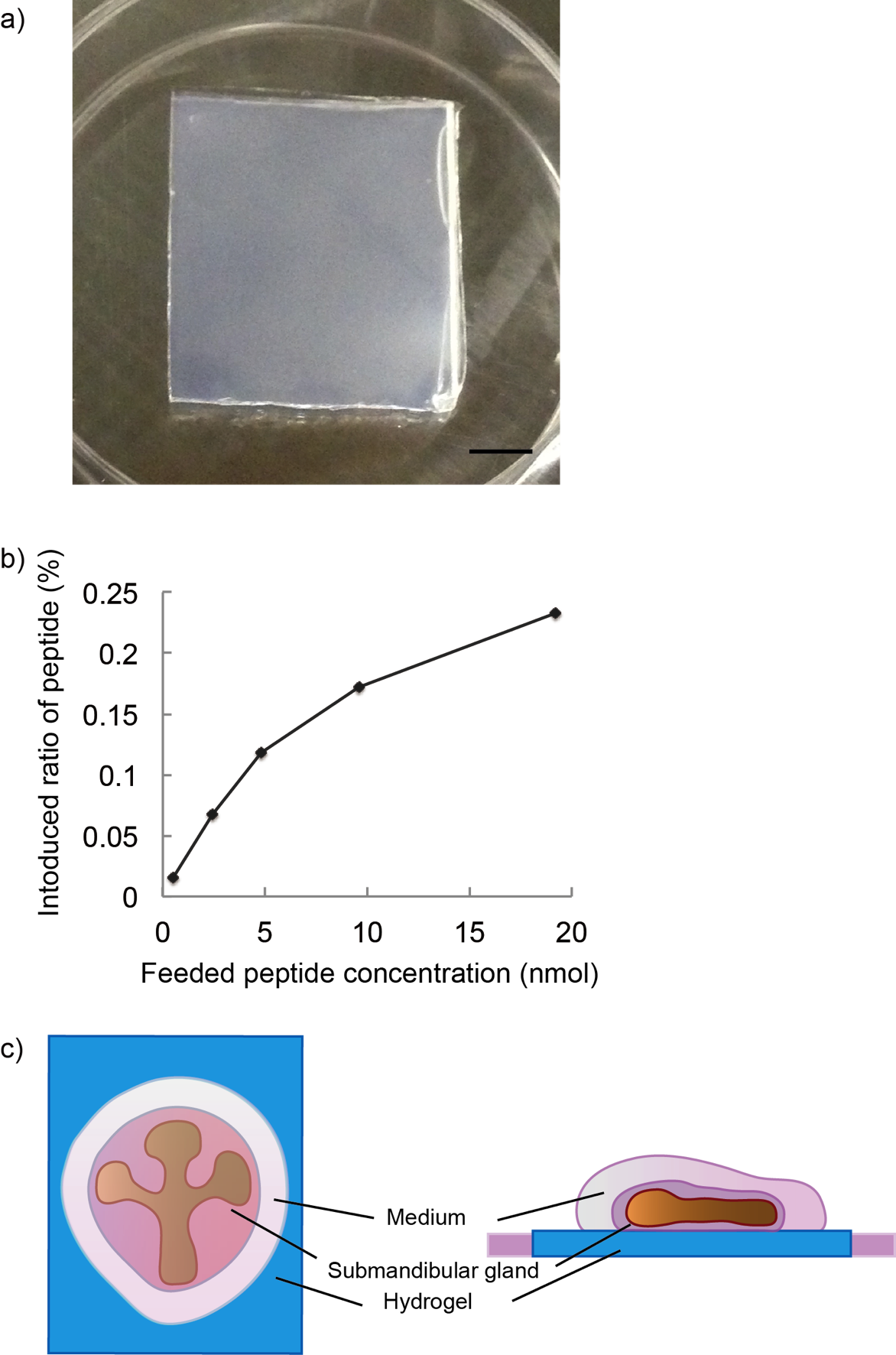


Figure S1

a) Alginate hydrogel sheet used for SMG tissue culture in this study (Bar = 2 mm). b) The ratio of RGD peptide introduced in alginate solution. c) Schematic illustration of SMG tissue culture system used in this study.

a)


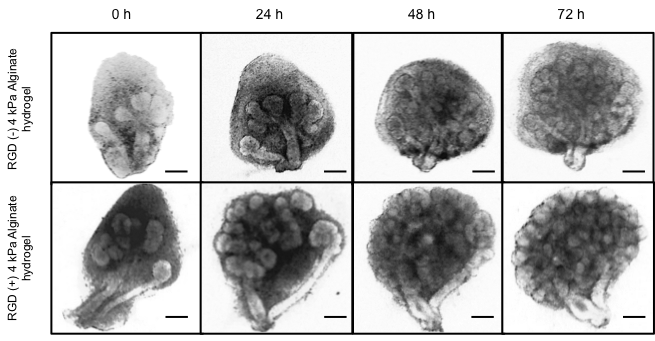


b)

*

*


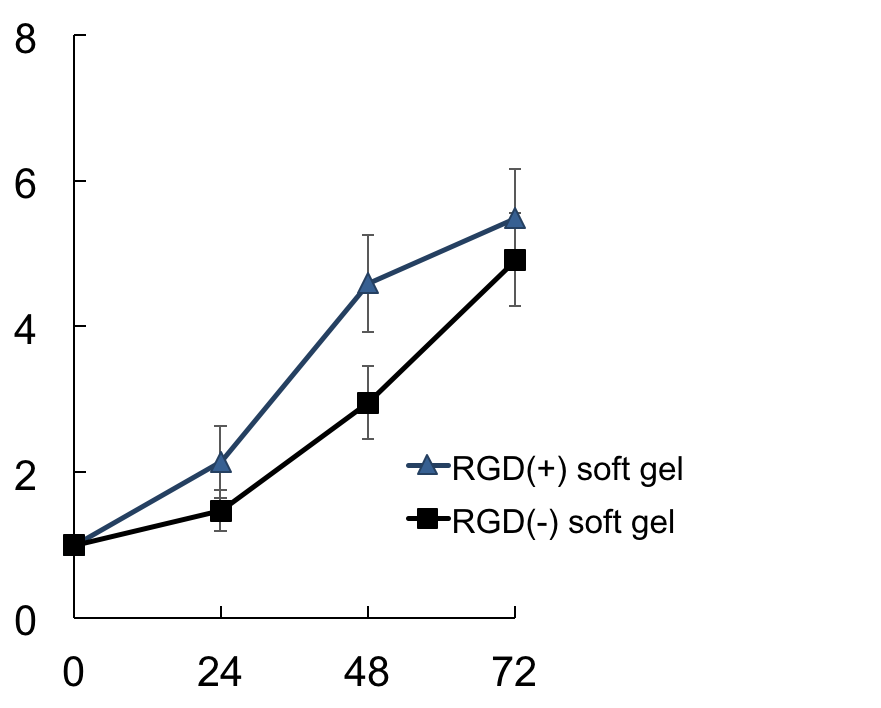


Time (h)


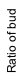


Figure S2

SMG cultured on 4 kPa Alginate hydrogel sheet with RGD modification showed higher bud expansion than that cultured on non-modified soft substrate (Bar = 100 µm)


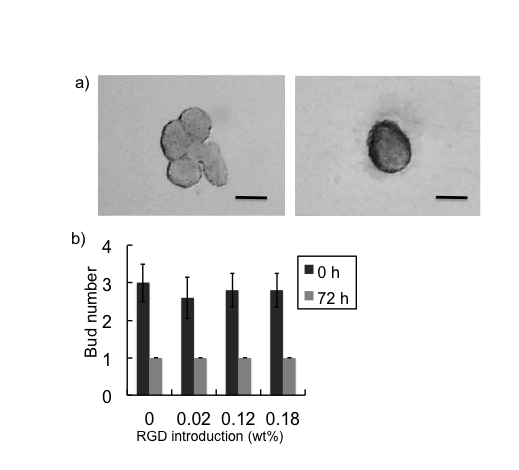


Figure S3

a) Isolated and cultured epithelial tissue (left: 0h, right: 72h, Bar = 50 μm). b) Bud number decreased to 1 when isolated epithelial tissue was cultured on RGD-modified gel sheets for 72 h.


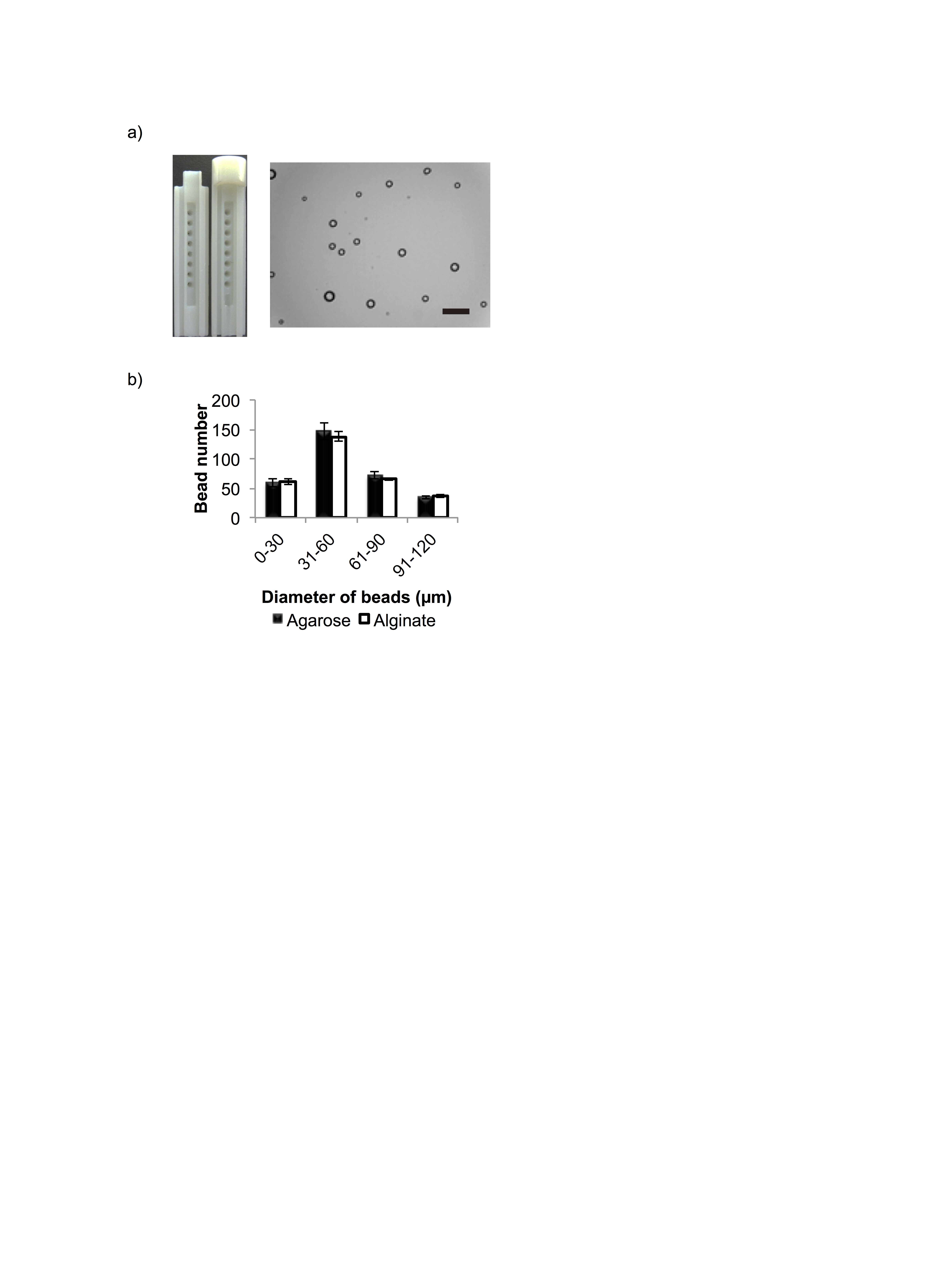


Figure S4

a) Original device (fabricated by 3D printing system) for hydrogel-bead synthesis (Bar=100 µm). b) Both agarose and alginate hydrogel beads with similar shape and size can be fabricated by using this device.


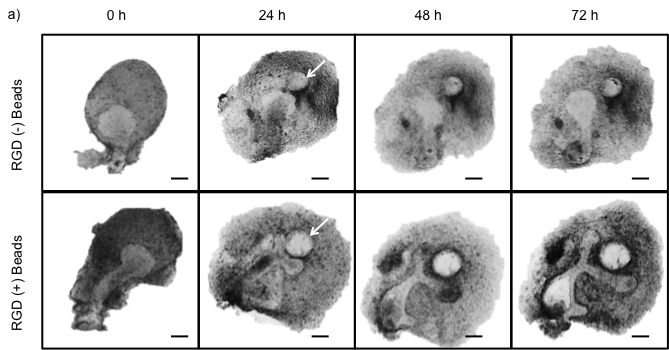


Figure S5

a) SMG cultured on the stiffer hydrogel with RGD (-) /RGD (+) beads (arrow) showed that RGD (+) bead modulate the SMG morphogenesis whereas, RGD (-) bead doesn’t have local effect on SMG branching morphogenesis (Bar=100 µm).

**Supplementary T**able 1

| Antibodies | Clonality | Supplier | Dilution | |
| --- | --- | --- | --- | --- |
|  |  |  | Immunohistochemistry | Western blot |
| FITC-conjugated Peanut agglutinin (PNA) | - | Sigma-Aldrich, MO | 1:200 | - |
| βIII-tubulin  (TUJ-1) | Mouse Monoclonal | R&D Systems, MN | 1:1000 | - |
| Anti-FGF7 | Rabbit polyclonal | AB Biotech, USA | 1:1000 | 1:1000 |
| Anti-FGF10 | Rabbit polyclonal | Millipore, USA | 1:2000 | 1:2000 |
| Anti-ki-67 | Rabbit polyclonal | Abcam, UK | 1:500 | - |
| ß-actin | Mouse monoclonal | Abcam, UK | - | 1:5000 |

List of the antibodies used in immunohistochemical
